# Novel Acetamide Indirectly Targets Mycobacterial Transporter MmpL3 by Proton Motive Force Disruption

**DOI:** 10.3389/fmicb.2018.02960

**Published:** 2018-12-04

**Authors:** Annanya Shetty, Zhujun Xu, Umayal Lakshmanan, Jeffrey Hill, Meng Ling Choong, Shu-Sin Chng, Yoshiyuki Yamada, Anders Poulsen, Thomas Dick, Martin Gengenbacher

**Affiliations:** ^1^Department of Medicine, Yong Loo Lin School of Medicine, National University of Singapore, Singapore, Singapore; ^2^Antimicrobial Drug Discovery Laboratory, Department of Microbiology and Immunology, Yong Loo Lin School of Medicine, National University of Singapore, Singapore, Singapore; ^3^Department of Chemistry, National University of Singapore, Singapore, Singapore; ^4^Experimental Therapeutics Center, A*STAR, Singapore, Singapore; ^5^Singapore Center for Environmental Life Sciences Engineering, Nanyang Technological University, Singapore, Singapore; ^6^Public Health Research Institute, New Jersey Medical School, Rutgers, The State University of New Jersey, Newark, NJ, United States

**Keywords:** *Mycobacterium tuberculosis*, *iniBAC*, cell envelope stress, flippase, high throughput screen

## Abstract

To identify novel inhibitors of *Mycobacterium tuberculosis* cell envelope biosynthesis, we employed a two-step approach. First, we screened the diverse synthetic small molecule 71,544-compound Enamine library for growth inhibitors using the non-pathogenic surrogate *Mycobacterium bovis* BCG as screening strain and turbidity as readout. Second, 16 confirmed hits were tested for their ability to induce the cell envelope stress responsive promoter p*iniBAC* controlling expression of red fluorescent protein in an *M. bovis* BCG reporter strain. Using a fluorescence readout, the acetamide E11 was identified. Resistant mutant selection and whole genome sequencing revealed the mycolic acid transporter Mmpl3 as a candidate target of E11. Biochemical analysis using mycobacterial spheroplasts and various membrane assays suggest that E11 indirectly inhibits MmpL3-facilitated translocation of trehalose monomycolates by proton motive force disruption. E11 showed potent bactericidal activity against growing and non-growing *M. tuberculosis*, low cytotoxic, and hemolytic activity and a dynamic structure activity relationship. In addition to activity against *M. tuberculosis*, E11 was active against the non-tuberculous mycobacterium *M. abscessus*, an emerging opportunistic pathogen. In conclusion, we identified a novel bactericidal anti-mycobacterial lead compound targeting MmpL3 providing an attractive starting point for optimization.

## Introduction

*Mycobacterium tuberculosis* (Mtb), the world's most successful pathogen, is responsible for an estimated 1.3 million deaths and 10.4 million new tuberculosis (TB) infections every year (WHO, 2017). The number of TB incidents has been marginally declining over the past decade but the burden of drug-resistant TB has increased to 600,000 cases reported in 2016 (WHO, 2017). The continuous rise and spread of drug-resistance, threatening global public health, renders development of new chemotherapeutic agents a top priority of TB control efforts to complement or substitute existing drug regimens.

The cell envelope of mycobacteria is a unique composition of carbohydrates and complex lipids which contribute to pathogenicity and set the genus *Mycobacterium* apart from other prokaryotes (Alderwick et al., 2015). It plays a critical role during infection by protecting intracellular (pathogenic) mycobacteria from the harsh environment of the phagosomal compartment (Gengenbacher and Kaufmann, 2012) and it acts as a permeability barrier for antibiotics in non-replicating nutrient-starved Mtb (Sarathy et al., 2013). Various topographies have been proposed for the cell envelope of mycobacteria with the most widely accepted model introducing a schematic division into three subdomains, the outer capsule, the tripartite cell wall consisting of the outer membrane (OM) bound to arabinogalactan-peptidoglycan complex, and the inner membrane (IM) (Daffé and Marrakchi, 2017). Roughly 10% of the Mtb genome is functionally devoted to the cell wall including a large number of genes essential for growth (Sassetti et al., 2003). It is therefore not surprising that several TB drugs in use or in development target essential biosynthetic pathways of cell wall components: (i) Mycolic acids: isoniazid (INH) (Ramaswamy et al., 2003), delamanid (Matsumoto et al., 2006; Sasaki et al., 2006), pretomanid (Manjunatha et al., 2009), and ethionamide (Vale et al., 2013); (ii) Arabinogalactans: ethambutol (EMB) (Forbes et al., 1962), the ethylenediamine SQ109 (Bogatcheva et al., 2010), and the benzothiazinones BTZ043 and PBTZ169 (Makarov et al., 2009); (iii) Peptidoglycans: cycloserine (Prosser and de Carvalho, 2013). INH and EMB have been part of the TB standard-of-care chemotherapy for more than 50 years demonstrating that mycobacterial cell envelope biosynthesis is a clinically validated intervention level and thus attractive for current drug discovery (Bhat et al., 2017).

Transcriptome analysis of Mtb cultures treated with INH identified three highly induced genes, *iniB, iniA*, and *iniC* (Alland et al., 1998). All three genes are organized in a single operon controlled by the *iniBAC* promoter which is upregulated by a broad range of mycobacterial cell envelope inhibitors (Alland et al., 2000). Due to its potent response to antibiotic-induced cell envelope stress, the *iniBAC* promoter in conjunction with firefly luciferase or *E. coli* β-galactosidase was utilized as reporter to identify inhibitors targeting the cell envelope (Alland et al., 2000).

Starting off from a diverse synthetic library of 71,544 small compounds, we performed a two-step whole cell screening campaign consisting of a growth inhibition assay with turbidity readout followed by evaluation of hits in a p*iniBAC* reporter assay detecting cell envelope stress. One hit capable of inhibiting mycobacterial growth and inducing cell envelope stress was profiled in depth including anti-mycobacterial activities, cytotoxicity, structure-activity relationship, mechanism of drug resistance, and mechanism of action.

## Materials and Methods

### Bacterial Strains, Cell Lines, Media, and Drugs

Mtb H37Rv (ATCC #27294), *M. bovis* BCG Pasteur (ATCC #35734) *M. smegmatis* (ATCC #700084), *M. avium* (ATCC #35717), *M. abscessus* (ATCC#19977), and the *M. abscessus* bamboo clinical isolate (Yee et al., 2017) were cultured in Middlebrook 7H9 broth supplemented with 0.05% Tween-80, 0.4% glycerol, and 10% albumin-dextrose-catalase enrichment (Becton Dickinson) at 37°C and 80 rpm or on Middlebrook 7H11 agar containing 0.2% glycerol and 10% oleic-acid-albumin-dextrose-catalase enrichment at 37°C. Mtb 18b (Stewart Cole, EPFL, Switzerland) was grown in the presence of 50 μg/ml streptomycin (STM) as previously described (Zhang et al., 2012). *Escherichia coli* (ATCC #25922) and *Staphylococcus aureus* (ATCC #29213) were maintained in LB broth (Becton Dickinson) at 37°C and 200 rpm.

The cell lines HepG2 (ATCC #HB8065), THP-1 (ATCC #TIB-202) and Vero (ATCC #CCL-81) were cultured in Dulbecco's modified Eagle's medium (DMEM) (Gibco) supplemented with 10% heat-inactivated fetal bovine serum (Gibco) and 2 mM glutamine (Gibco) in 5% humidified CO_2_ at 37°C. Red blood cells (RBCs) were obtained from the Interstate Blood Bank Inc. laboratory, USA.

A chemically diverse screening library of 71,544 drug-like compounds was procured from Enamine, USA. Analogs of E11 were obtained from the compound collection of the Experimental Therapeutics Center (A^*^Star, Singapore). All other antibiotics and chemicals were purchased from Sigma-Aldrich. Drugs were dissolved in 90% dimethyl sulfoxide, filter-sterilized using 0.22 μm polytetrafluoroethylene membrane filters and stored at −20°C until use.

### High Throughput Screening of Enamine Library

Primary screening was carried out by the Experimental Therapeutics Center (A^*^Star, Singapore) using exponentially growing *M. bovis* BCG diluted to OD_600_ of 0.1 in glycerol-free Middlebrook 7H9 medium and flat bottom clear 384-well plates (Greiner Bio-One) sealed with breathable membranes (Breath Easy, Sigma-Aldrich). Compounds were screened in duplicates at a final concentration of 12.5 μM (40 μl/well) using turbidity as single-point readout after 3 days of incubation at 37°C. The TB first line drug rifampicin at 10 μM served as positive control. All assay wells including control wells had a final concentration of 0.6% DMSO. A pilot screen of 1,000 compounds carried out prior the main campaign confirmed robustness and reproducibility of the assay (signal-to-noise ratio = 5.6 ± 0.3; Z' factor = 0.8 ± 0.1) (Zhang et al., 1999).

### Construction of *M. bovis* BCG P*iniBAC*-mCherry Reporter Strain

The 191 bp promoter region upstream of the *iniBAC* operon was amplified by polymerase chain reaction from *M. bovis* BCG DNA using 5′-GCGGCCGCTAAGTTCCGGACCGGCGTA-3′ and 5′-CCGGGATCCCTTCATTTCCCTTCAATAGAAGA-3′ and the mCherry gene was amplified from pGMEH-P38-mRFP (Addgene #27058) using 5′-CCGGGATCCATGGTGAGCAAGGGCGAGG-3′ and 5′-CCGGAATTCCTACTTGTACAGCTCGTCCAT-3′. Amplicons were purified, digested with *Not*I/*BamH*I or *BamH*I/*EcoR*I and cloned into the integrative shuttle vector pMV306 (Stover et al., 1991) by using the *Not*I and *EcoR*I restriction sites. The reporter plasmid pMV306-p*iniBAC*-mCherry was verified by automated sequencing prior electroporation into *M. bovis* BCG. Transformants were selected on agar containing 25 μg/ml kanamycin. The final recombinant reporter strain, BCG-p*iniBAC*-mCherry was used for the secondary screen.

### p*iniBAC* Reporter Assay

Exponentially growing BCG-p*iniBAC*-mCherry was seeded into 96-well plates at an OD_600_ of 0.2 and incubated at 37°C for 24 h in the presence of test compound. The increase in mCherry fluorescence signal over 24 h assay time was measured using an M200Pro plate reader instrument (Tecan; excitation λ = 587 nm, emission λ = 630 nm) and expressed as relative fluorescence units (RFU). Dose response induction was assessed by 2-fold serial dilutions of the compounds from 100 μM. The p*iniBAC* reporter assay was validated by anti-mycobacterial cell wall inhibitors (INH; ethambutol, EMB; ethionamide, ETH) and non-cell wall inhibitors (ciprofloxacin, CIP; bedaquiline, BDQ; STM).

### Determination of Minimal Inhibitory Concentration and Bactericidal Activity Against *M. tuberculosis*

Minimum inhibitory concentrations (MICs) were determined by the broth microdilution method (Wiegand et al., 2008). Exponentially growing precultures were seeded in clear 96-well flat-bottom plates (Greiner Bio-One) at OD_600_ = 0.05 in the presence of two-fold serial dilutions of assay compounds in a volume of 200 μl/well. Assay plates were sealed (Breath-Easy membrane, Sigma-Aldrich) and incubated for 7 days at 37°C and 80 rpm prior turbidity determination (OD_600_, Tecan M200Pro plate reader). MIC curves were plotted using the Graph Pad Prism 5 software and the concentration that inhibits 50 and 90% of growth compared to the drug-free control were defined as MIC_50_ and MIC_90_, respectively. To establish the Minimum bactericidal concentration (MBC), the colony forming units (CFUs) per assay well were determined by plating serial dilutions of samples onto agar (Murugasu-Oei and Dick, 2000). Colonies were counted after 3–4 weeks of incubation at 37°C. The MBC was defined as the lowest concentration of test compound that killed 90% of the initial inoculum.

### Determination of Bacterial Activities Against Non-Replicating *M. tuberculosis*

Non-replicating Mtb H37Rv and streptomycin auxotroph Mtb 18b were generated as previously described by nutrient deprivation or streptomycin starvation (Gengenbacher et al., 2010; Zhang et al., 2012). Non-replicating bacilli were seeded in 14 ml round bottom tubes at OD_600_ of 0.1 (nutrient-starved Mtb H37Rv) or of 0.2 (streptomycin-starved Mtb 18b) and incubated with designated concentrations of drugs for 10 days at 37°C and 80 rpm. Serial dilutions of samples were then plated onto agar. To allow for growth of the Mtb 18b, respective agar was supplemented with 50 μg/ml STM. Colonies were counted after 4–5 weeks of incubation at 37°C.

### Determination of MICs Against *M. smegmatis, M. abscessus, M. avium, E. coli*, and *S. aureus*

Exponentially growing cultures of *M. smegmatis, M. abscessus*, and *M. avium* were diluted to OD_600_ of 0.005 and incubated in clear 96-well flat-bottom microtiter plates in the presence of test drug for 1, 3, and 4 days, respectively prior turbidity readout. *S. aureus* and *E. coli* were diluted to OD_600_ of 0.005 and incubated overnight with test drugs. Ten-point two-fold serial dilutions of test compounds starting from 100 μM were used.

### Resistant Mutant Selection and Whole Genome Sequencing

Spontaneous resistance mutants were selected by plating 10^8^ exponentially growing *M. bovis* BCG bacilli on agar containing 4 × and 8 × MIC_90_ of test drugs. After 4–6 weeks drug resistance was verified by re-streaking single colonies on agar containing similar concentration of drugs. 10 ml cultures of individual clones were grown to the mid-log phase and used for DNA isolation and whole genome sequencing (Yee et al., 2017) using Covaris shearing and Illumina TruSeq nano DNA library preparation followed by sequencing on an Illumina MiSeq platform (AITbiotech, Singapore). Mutations observed in the *mmpL3* gene were confirmed by automated sequencing of targeted amplicons generated by specific oligonucleotides 5′- CCGGAATTCGAGTGTTCGCCTGGTGGGGTC-3′, 5′-CTACGACACCGAGACGG CAGTA-3′, 5′-CCGAAGCTTTTAAAGGCGTCCTTCGCGGC-3′ and 5′-TGGCTGCCGTCGTCGTAGAA- 3′.

### Determination of Cytotoxicity, Hemolytic Activity and Microsomal Stability

The cell lines HepG2 (Human liver carcinoma), THP-1 (Human monocytic leukemia), and Vero (African green monkey kidney epithelia) were used to evaluate cytotoxicity of test drugs. Cells were grown to 80% confluency and seeded on tissue culture-treated 96-well microtiter plates at 50,000 cells/well (HepG2, Vero) or 20,000 cells/well (THP-1). Two-fold serial dilutions of drugs were used starting from 100 μM. Serial dilutions of 0.1% Triton X-100 served as positive control. After 24 h of incubation at 37°C and 5% CO_2_, media was carefully aspirated and replaced with media containing 33 μg/ml [3-(4,5-dimethylthiazol-2-yl)-5-(3-carboxymethoxyphenyl)-2-(4-sulfophenyl)-2H-tetrazolium inner salt (MTS solution, Promega). Viability assay plates were incubated for 2 h at 37°C/5% CO_2_ prior absorbance measurement at 490 nm wavelength. For determination of hemolytic activity 1,000,000 RBCs were incubated in the presence of test drugs or 0.1% Triton X-100 in 200 μl of saline (96-well format) and incubated for 24 h at 37°C. RBCs were then pelleted by centrifugation (300 × *g*) and absorbance of supernatants were measured at λ = 540 nm. Cytotoxic concentration_50_ (CC_50_) and hemolytic concentration_50_ (HC_50_) were defined as concentration that lysed 50% of cells. The selectivity index (SI) was calculated as ratio of CC_50_/MIC_50_ or HC_50_/MIC_50_. Microsomal stability was determined using rat liver microsomes (Sigma Aldrich) as described elsewhere (Yang et al., 2017).

### Membrane Potential and Permeability Determination

The impact of drugs on the polarization of the mycobacterial membrane was studied using the lipophilic cationic dye, 3,3-diethyloxacarbocyanine iodide (DiOC_2_) that has membrane potential-dependent partitioning across energized biological membranes as described earlier (Novo et al., 2000; Mukherjee et al., 2016). Membrane permeability as a surrogate of viability was assessed using the SYTO 9/propidium iodine-based (Lebaron et al., 1998) Live/Dead *Bac*Light bacterial viability kit according to the manufacture's protocol (Thermo Fisher).

### TMM Accessibility to LysB Degradation Assay, Whole Cell Lipid Profiling and Determination of Intracellular pH

*M. smegmatis* spheroplasts were used to evaluate putative MmpL3 inhibitors (Xu et al., 2017). Briefly, spheroplasts were pre-treated with indicated concentrations of E11 for 15 min, and metabolically labeled with sodium [1-^14^C]-acetate (0.2 μCi/ml, Perkin Elmer) for 2 h. DMSO serves as a negative control and BM212 serves as a positive control. Subsequently, 1-ml spheroplasts were aliquoted into separate microcentrifuge tubes and treated with purified LysB (50 μg/ml) for 30 min at 37°C. Lipids were extracted from harvested spheroplast cells. The [^14^C]—counts were measured using scintillation counting (MicroBeta2®, Perkin-Elmer) and taken as the levels of total lipids isolated from the spheroplasts. Equal amounts of radioactivity were spotted onto Silica 60 F_254_ thin layer chromatography (TLC) plates (Merck), separated using a chloroform-methanol-water (30:8:1) solvent system and visualized via phosphor imaging (STORM, GE Healthcare). Signal of individual lanes were quantified by the ImageQuant TL analysis software v7.0 (GE Healthcare).

Both *M. smegmatis* and *M. bovis* BCG were used to test E11 in inhibiting the TDM production. Briefly, *M. smegmatis* and *M. bovis* BCG (OD = 0.3) were incubated with indicated concentrations of E11 for 1 h at 37°C, following by sodium [1-^14^C]-acetate (0.2 μCi/ml) labeling for 1 h and 3 h. DMSO and ethambutol serve as negative controls and BM212 serves as a positive control. Total lipids of *M. smegmatis* and *M. bovis* BCG were processed using a similar extraction and analysis procedure.

The effects of inhibitors on ΔpH were determined using BCECF, a pH-sensitive fluorescent dye activated inside cells via esterase-mediated hydrolysis of BCECF-AM. First, a standard curve was generated to show the correlation of fluorescence (λ_ex_ 488 nm/λ_ex_ 440 nm) values and *M. smegmatis* spheroplasts in various pH buffers (6.0–8.0). To test the effects of putative MmpL3 inhibitors on intracellular pH (and hence ΔpH), spheroplasts (OD = 0.8) in 1 × SMM buffer at pH 6.8 were pre-treated with indicated concentrations of E11, and incubated at 37°C for 30 min. DMSO and BM212 was used as negative controls, while CCCP was used as a positive control. 20 μM BCECF-AM was then added to the samples and incubation was continued for 30 min before fluorescence measurements. Fluorescence excitation profiles (λ_ex_ 488 nm/λ_ex_ 440 nm) of BCECF for each condition were averaged (across three technical replicates) and calibrated against the standard curve.

### Statistical Analysis

Statistical analysis for comparison of various experimental conditions with control groups was performed using Student's *t*-test with significance set at *p* < 0.05.

### Biosafety and Biosecurity

The research reported here has been reviewed and approved by the Institutional Biosafety Committee of the National University of Singapore.

## Results

### Primary Growth Inhibition Screen Delivers 16 Confirmed Hits

**Figure 1:**
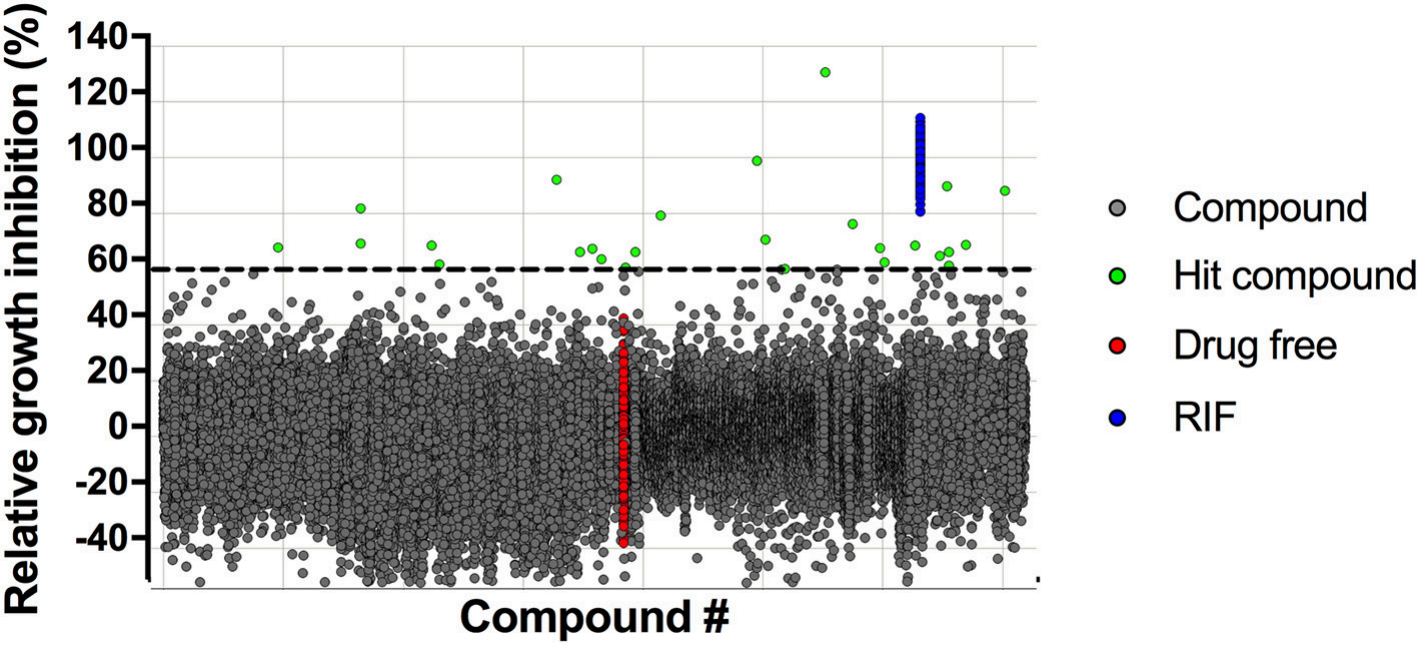
Scatterplot of high throughput growth inhibition screen. The Enamine library of 71,544 chemically diverse drug-like compounds were tested at a single-point concentration of 12.5 μM in 384-well format for growth inhibitors of *M. bovis* BCG using turbidity as read-out. Results are expressed as growth inhibition of individual compounds relative to the TB first line drug rifampicin that served as positive control and was set to 100% inhibition. The cut-off for hit selection was 60%. Of the 26 primary hits, 16 hits could be confirmed in dose-response experiments (Table 1).

A robust single-point *M. bovis* BCG screening assay in 384-well format and simple turbidity measurement as readout was used to screen the Enamine library of 71,544 synthetic small molecule compounds for mycobacterial growth inhibitors in duplicates at a concentration of 12.5 μM. Compounds achieving growth inhibition of at least 60% compared to the drug-free control were defined as hits (Figure 1). Of the 26 primary hits, 16 re-supplied solids were confirmed by the same single-point growth inhibition assay and showed a dose response profile with MIC_50_ <50 μM (Table 1).

**Table 1:** *In vitro* potency of primary mycobacteria growth inhibitor screen hits.

**Compound**	**Structure**	MIC_50_ (μM)^*^	**MIC_90_ (μM)**	**Compound**	**Structure**	**MIC_50_ (μM)**	**MIC_90_ (μM)**
E01	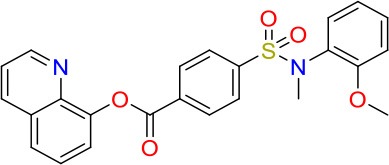	6.25	6.25	E09	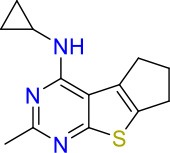	12.5	50
E02	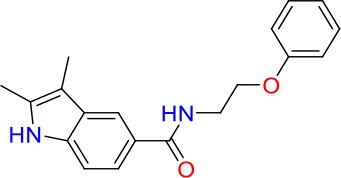	3	50	E10	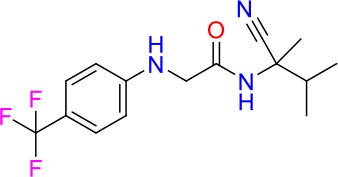	8	25
E03	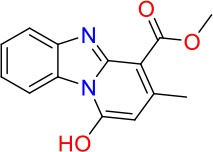	50	>100	E11	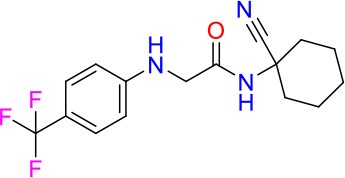	8	12.5
E04	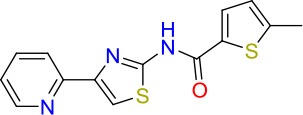	20	>100	E12	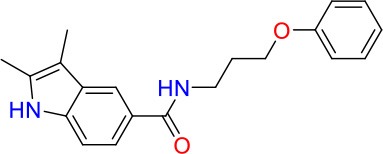	12.5	>100
E05	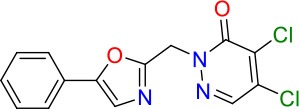	8	25	E13	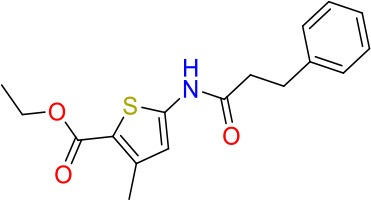	30	>100
E06	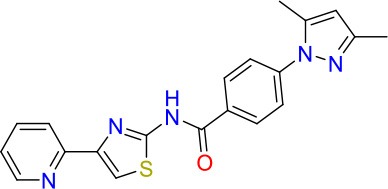	25	>100	E14	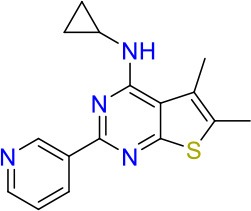	40	>100
E07	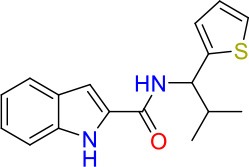	1.25	6.25	E15	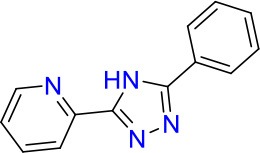	20	100
E08	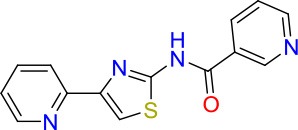	20	100	E16	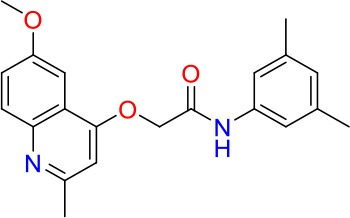	0.8	25

* *Minimum Inhibitory Concentrations (MICs) are defined as drug concentration that inhibit 50% (MIC_50_) or 90% (MIC_90_) of growth relative to the drug-free control*.

### Secondary Screen for Growth Inhibitors Identifies Two Cell Envelope Stress-Inducing Hits

**Figure 2:**
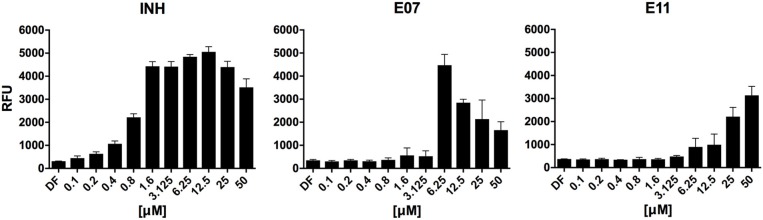
Hit compounds E07 and E11 induce cell wall stress in *M. bovis* BCG. A reporter strain expressing red fluorescent protein under control of the cell wall stress-sensitive *iniBAC* promoter was incubated with various concentrations of each test drug or the TB first line cell wall inhibitor isoniazid (INH). The fluorescence signal was measured after 24 h incubation and expressed as Relative Fluorescence Units (RFU). Shown are means and standard deviations of one representative experiment out of three biological replicates.

To identify hits targeting cell envelope biosynthesis, we constructed a BCG reporter strain expressing red fluorescent protein (RFP) under the control of the *iniBAC* promoter. Mycobacterial cell envelope inhibitors were previously shown to induce the *iniBAC* operon (Alland et al., 2000). We confirmed specificity of our BCG *iniBAC*-RFP reporter by testing 29 anti-mycobacterials including a range of known cell envelope inhibitors (Supplementary Figure 1) at a single point of 1 × MIC_90_. Drugs directed at peptidoglycan biosynthesis had a dichotomous induction pattern; β-lactams showed moderate to strong induction and bacilli lysing drugs including cycloserine and vancomycin failed to upregulate the *iniBAC* reporter. We then subjected the 16 confirmed hits of the primary growth inhibition screen (Table 1) at different concentrations to the BCG *iniBAC*-RFP reporter assay. Compounds E07 and E11 showed a concentration-dependent induction of the reporter indicating cell envelope stress (Figure 2). A drop of fluorescence signal was observed at high concentrations of INH and E07 (Figure 2). Plating these samples on agar confirmed that cell death was the underlying cause rather than a decreased relative reporter induction per cell. E07, an analog of an indolecarboxamide compound series with demonstrated anti-mycobacterial activity, was not further pursued (Kondreddi et al., 2013). The acetamide E11 was taken forward and characterized in depth.

### E11 Resistant Mutant Selection and Whole Genome Sequencing Suggest MmpL3 as Possible Target

Identification of the cellular target of an inhibitor is critical to elucidate the mechanism of action and the mechanism of resistance. We selected for spontaneous resistant *M. bovis* BCG mutants on agar containing various concentrations of E11. Three mutants isolated in three different experiments showed >10-fold increase in resistance to E11 compared to *M. bovis* BCG wildtype, having an MIC_90_ > 100 μM (Supplementary Figure 2). The observed mutation frequency of E11 was 1 × 10^−8^ which is comparable to RIF (1 × 10^−8^) but two orders of magnitude lower than INH (1 × 10^−6^) (Rao et al., 2013). Whole genome sequencing of the three E11 *M. bovis* BCG mutants identified a single nucleotide polymorphism in the *mmpL3* gene at position 1927 (G → A). The mutation leads to an amino acid change of V643 to M located in transmembrane domain 10 of the mycolic acid transporter MmpL3 (Belardinelli et al., 2016) (Supplementary Figure 3) suggesting that this essential flippase could be a cellular target of inhibitor E11.

### E11 Indirectly Blocks Translocation of Trehalose Monomycolates across the IM

**Figure 3:**
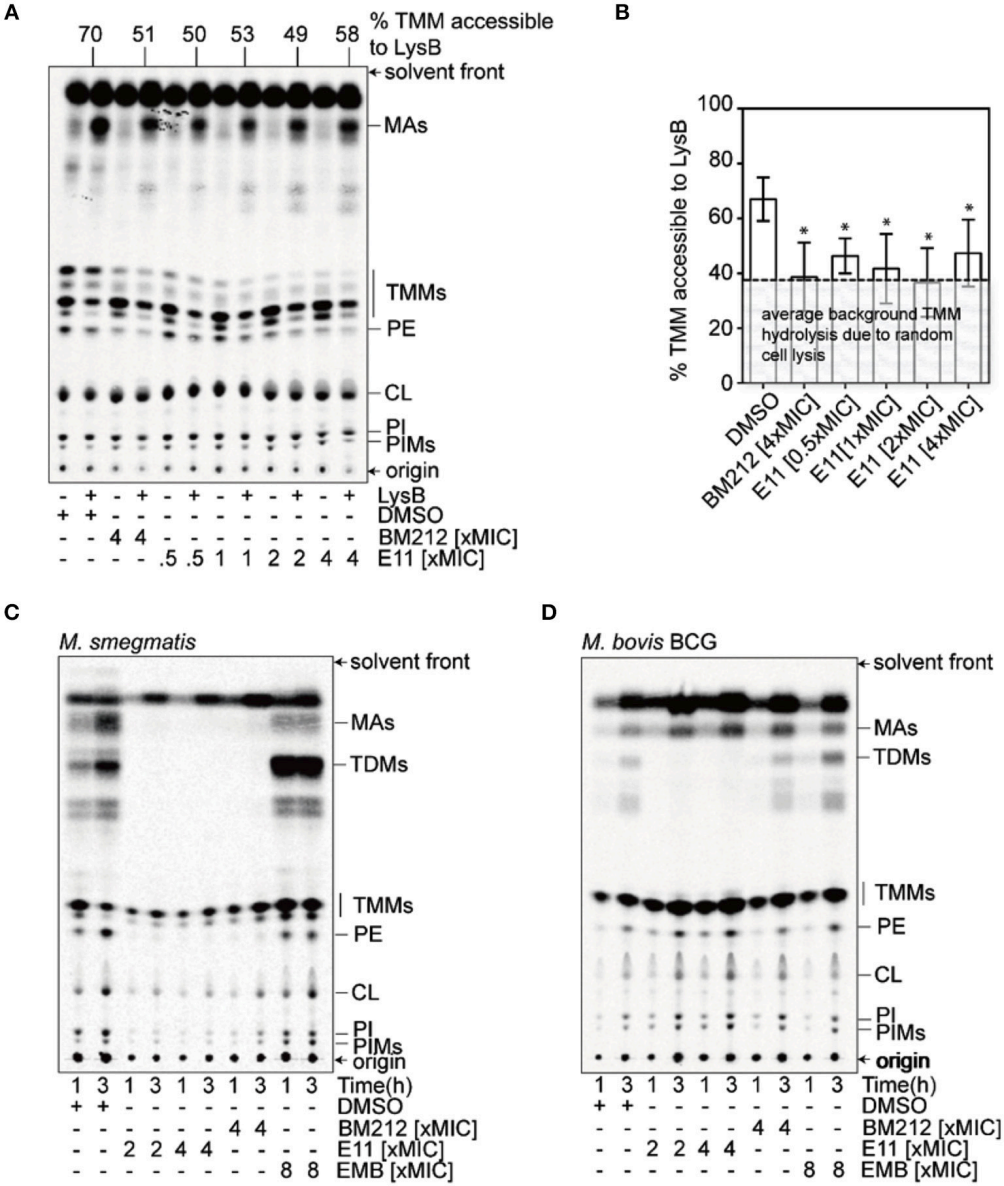
E11 reduces the TMM translocation across the IM and inhibits TDM production in mycobacteria. **(A)** Representative TLC analyses of [^14^C]-labeled lipids newly-synthesized in the presence of indicated concentrations of BM212 and E11, and extracted from *M. smegmatis* spheroplasts following treatment with or without purified LysB. DMSO was used to dissolve the respective compounds and thus serve as negative controls. Equal amounts of radioactivity were spotted for each sample. **(B)** A graphical plot showing the effects of various compounds on the amounts of LysB-accessible TMMs in spheroplasts. The percentage of TMMs accessible to LysB is given by the difference in TMM levels between samples with or without LysB treatment, normalized against that in control samples without LysB treatment. TMM levels in each sample were quantified as a fraction of total mycolates (TMM+MA). Average percentages and standard deviations from three biological replicates are plotted. The average background of TMM hydrolysis due to random cell lysis during the experiment (~38%) is indicated (Supplementary Figure 5). Student's *t*-test: ^*^p < 0.05 compared to the DMSO control. **(C,D)** TLC analysis of newly-synthesized [^14^C]-labeled lipids extracted from *M. smegmatis* **(C)** and *M. bovis* BCG **(D)**, visualized by phosphor imaging. Bacilli were treated with indicated concentrations of BM212, and E11 for 1 h prior to the radiolabeling, and harvested at designated time points followed by lipid extraction. Ethambutol (EMB), which is an arabinogalactan synthesis inhibitor, serves as a negative control. The developing solvent system comprises chloroform-methanol-water (30:8:1). TDM, trehalose dimycolate; TMM, trehalose monomycolate; MA, mycolic acid; PE, phosphatidylethanolamine; CL, cardiolipin; PI, phosphatidylinositol; PIM, phosphatidylinositol mannoside.

MmpL3 is a transporter consisting of 12 transmembrane domains (Belardinelli et al., 2016). It translocates mycolic acids synthesized in the cytosol across the IM, where these mycobacteria-specific building blocks get subsequently transported and incorporated in the OM (Xu et al., 2017). To evaluate whether E11 indeed inhibits MmpL3, we used a trehalose monomycolate (TMM) topological assay in *M. smegmatis* spheroplasts (Xu et al., 2017), cells devoid of the OM and cell wall (Dhiman et al., 2011) but with intact IM (Udou et al., 1983). The biological activity of the flippase MmpL3 in spheroplasts can be assessed by specific lipases that can only access mycolic acids that have been flipped to the outer leaflet of the IM (Xu et al., 2017). We monitored accessibility of newly synthesized TMM to the lipase lysin B (LysB) in spheroplasts treated with E11 and profiled extracted lipids by thin layer chromatography. The MmpL3 inhibitor BM212, a 1,5-diarylpyrrole, served as a positive control (La Rosa et al., 2012). We found that E11 significantly reduced LysB-accessibility of TMM without clear dose dependence (Figures 3A,B). Consistent with this observation, E11 completely abolished the conversion of TMM to trehalose dimycolate (TDM) in whole *M. smegmatis* and *M. bovis* BCG cells (Figures 3C,D). E11 did not impact LysB activity excluding direct interference with our assay (Supplementary Figure 6). Overall, these results suggest that E11 can inhibit flipping of TMM by MmpL3. While E11 did not affect the membrane potential (Supplementary Figure 7), it disrupted the proton gradient across the IM (Table 3). We conclude that E11 indirectly blocks TMM translocation by preventing MmpL3 from utilizing the proton motive force for transport.

### E11 Shows Potent Bactericidal Activity Against Growing and Non-growing *M. tuberculosis* and Acceptable Cytotoxicity Profile

**Figure 4:**
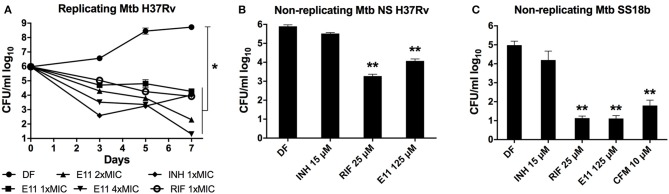
E11 kills replicating and non-replicating *M. tuberculosis* (Mtb). **(A)** Time-kill kinetics of E11 in growing Mtb at 1 ×, 2 ×, and 4 × MIC_90_ corresponding to 12.5, 25, and 50 μM concentrations, respectively. Isoniazid (INH) 1 × MIC_90_ 1.5 μM, rifampicin (RIF) 1 × MIC_90_ 0.025 μM. **(B)** Bactericidal activity of E11 on nutrient-starved (NS) non-replicating Mtb H37Rv (Gengenbacher et al., 2010) and **(C)** streptomycin-starved (SS) non-replicating Mtb 18b (Zhang et al., 2012). INH, RIF, and clofazimine (CFM) were used as controls. Each dataset is representative of two independent experiments with duplicate samples. Means and standard deviations are shown. DF, drug free; CFU, colony forming unit. Student's *t*-test ^*^p < 0.05; ^**^p < 0.01 compared to DF control.

We defined *in vitro* potency of E11 by assessing the bactericidal activity of this acetamide against replicating and non-replicating Mtb. A decrease in viability over time was observed when growing Mtb cultures were exposed to 12.5, 25, and 50 μM corresponding to 1 ×, 2 ×, and 4 × MIC_90_ of E11 (Figure 4A). The time kill kinetics revealed dose-dependent killing of Mtb by E11 ranging from ~2 logs at 1 × MIC_90_ to >4 logs at 4 × MIC_90_ over the duration of the experiment (Figure 4A) and controls performed as previously reported (de Steenwinkel et al., 2010). Two established culture models were used to produce dormant bacilli by either nutrient deprivation of Mtb H37Rv laboratory strain or streptomycin starvation of the streptomycin auxotroph strain Mtb 18b (Gengenbacher et al., 2010; Zhang et al., 2012). E11 caused a significant viability drop in both culture models that reached 4 logs in streptomycin-starved Mtb 18b (Figures 4B,C). The much lower bactericidal activity of E11 on nutrient-starved Mtb at relatively high concentrations of 10 × MIC_90_ is consistent with previous reports on the notoriously high level of drug tolerance observed in this culture model (Xie et al., 2005; Gengenbacher et al., 2010). Although E11 was bactericidal for non-replicating bacilli, it did not affect the permeability or membrane potential of mycobacteria (Supplementary Figure 4), the latter consistent with results in spheroplasts (Supplementary Figure 7).

E11 also showed inhibitory activity (MIC_90_) in other mycobacterial species including *M. smegmatis* (50 μM), non-tuberculous *M. abscessus* ATCC (12 μM) and clinical isolate *M. abscessus* Bamboo (25 μM) (Yee et al., 2017) but was inactive in *M. avium* ATCC, Gram-positive *S. aureus* and Gram-negative *E. coli* with MIC_90_ >100 μM, suggesting specificity of E11 to most mycobacteria. To assess cytotoxicity of E11, we measured the impact of the compound on the viability of human cell lines HepG2 and THP-1 as well as on the primate cell line Vero. The hemolytic activity was determined using human red blood cells. In tested cell lines and red blood cells the selectivity index (SI) for E11 was >25 indicating low cytotoxicity. E11 was moderately stable in rat liver microsomes with a half-life time of 28.6 min and CL_int_ = 80.7 μl/min/mg of protein. In summary, E11 has potent bactericidal activity against growing and non-growing Mtb, acceptable cytotoxicity profile, and appeared to be mostly mycobacteria-specific.

### E11 Displays Dynamic Structure-Activity Relationship

The E11 lead compound contains a lipophilic CF_3_ substituent in *para* position of the aromatic ring, which when replaced with the polar cyano of derivative E11-2 (logP = 0.7) reduced Mtb activity by 10-fold. Likewise, compounds E11-5 and E11-6 with polar methyl-esters in the *meta* and *para* position were significantly less potent. In contrast, the hydrophobic dichloro-substitution at R_1_ and R_3_ resulted in compound E11-3 (logP = 2.3), which was equipotent to the lead (Table 2), suggesting important hydrophobic interactions with the target or improved penetration of the lipophilic analogs. The only outlier was compound E11-4 (logP = 2.5) with a trifluoro-methoxy *ortho* substituent, inactive most likely due to lack of room for substitution around the *ortho* position, since the same substituent in the *meta* and *para* position resulted in the most potent derivatives E11-7 and E11-10 (Table 2). Furthermore, replacing the cyclopentane ring of the lead with 3, 6, and 7-membered rings or an acyclic branched alkane, enhanced and/or maintained the lead's potency (compounds E11-7 to E11-10). Collectively, there seems to be a good correlation between lipophilicity (logP), decreased polarity (PSA), and growth-inhibitory activity (Table 2). Based on the structure-activity relationship study a preliminary pharmacophore can be deduced (Table 2). We then tested cross-resistance of the acetamide compound series (E11-2–E11-10) on the E11-resistant *M. bovis* BCG mutant C5 (Supplementary Figure 2). All derivatives showed a shift in MIC_50_ >50 μM demonstrating on-target activity (Supplementary Figure 8). Moreover, a correlation between *iniBAC*-RFP induction and potencies of the structural derivatives was observed, i.e., only analogs that had an MIC were able to trigger a cell envelope stress response (Supplementary Figure 9). For two compounds of the acetamide series, E11-7 and E11-9, we were able to obtain *M. bovis* BCG spontaneous resistance mutants. Targeted sequencing of the *mmpL3* gene revealed that E11-9 resistant mutants had the same mutation as mutants against the parent compound E11 while E11-7 resistance was conferred by a nucleotide T875C transition that resulted in an I292T amino acid change. In conclusion, the E11-derived acetamide compound series shows a dynamic structure-activity relationship and induces cell envelope stress by inhibiting mycolic acid transport.

**Table 2:** E11 and analogs.

**Compound**	**Structure**	MIC_50_ (μM)^*^	logP^*^	PSA^*^
E11	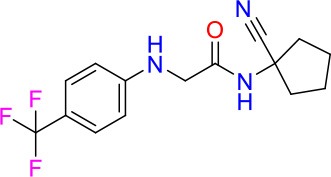	8	2.3	74
E11-2	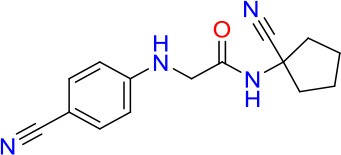	100	0.7	100
E11-3	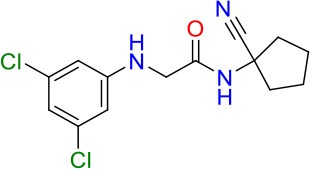	5	2.3	74
E11-4	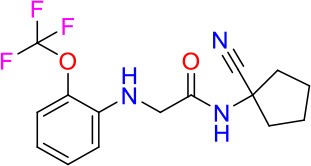	>100	2.5	81
E11-5	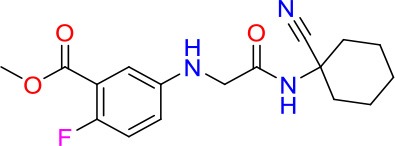	>100	1.7	110
E11-6	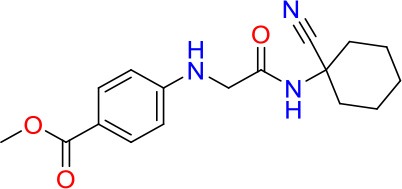	50	1.4	110
E11-7	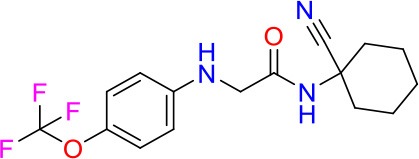	3	2.8	82
E11-8	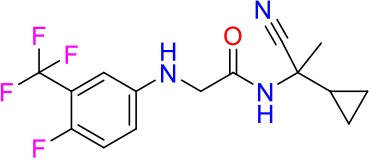	7	2.6	73
E11-9	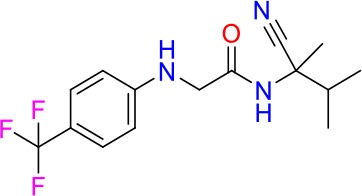	10	2.6	72
E11-10	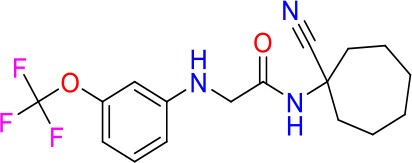	3	3.1	81

* *Minimum Inhibitory Concentration_50_ (MIC_50_), drug concentration that inhibits 50% of growth as compared to the drug-free control; logP, is the logarithm of partition coefficient between n-octanol and water; PSA, Polar Surface Area*.

**Table 3:** E11 affects the ΔpH across the IM in *M. smegmatis* spheroplasts.

**Drug**	**pH_outside_^a^**	**^b^pH_inside_**	**^c^ΔpH**
DMSO	6.80	7.17 (±0.02)	0.37 (±0.02)
4xBM212	6.80	7.12 (±0.01)	0.32 (±0.01)
0.5xE11	6.80	7.11 (±0.01)^*^	0.31 (±0.01) ^*^
1 × E11	6.80	7.01 (±0.01)^**^	0.21 (±0.01) ^**^
2 × E11	6.80	6.98 (±0.01)^**^	0.18 (±0.01) ^**^
4 × E11	6.80	6.83 (±0.01)^**^	0.03 (±0.01) ^**^
2 μM CCCP	6.80	6.77 (±0.01)^**^	−0.03 (±0.01) ^**^
5 μM CCCP	6.80	6.57 (±0.02)^**^	−0.23 (±0.02) ^**^

a *refers to pH of external buffer*.

b *intracellular pH obtained using the BCECF dye. Measurements of the fluorescence excitation profile (λ_ex_ 488 nm/λ_ex_ 440 nm) of BCECF were averaged (across the three biological replicates) and calibrated against a standard curve. Standard deviations are given in parenthesis*.

c pH_inside_ - pH_outside_

Student's t-test:

* p < 0.05;

** *p < 0.01 compared to the DMSO control*.

## Discussion

The cell envelope targeting TB first line drugs INH and EMB have been successfully in use for decades establishing the mycobacterial cell envelope as a clinically validated attractive drug target space. A robust number of novel cell envelope inhibitors is currently in the development pipeline raising hopes for new antimycobacterials equally potent as INH becoming available in the near future (Bhat et al., 2017).

Using a two-step approach consisting of whole cell high throughput screening for growth inhibitors of the Mtb-surrogate *M. bovis* BCG combined with a cell envelope stress reporter assay, we identified the novel acetamide E11 (Figures 1, 2). Spontaneous resistance mutant selected with E11 and whole genome sequencing revealed a mutation leading to an amino acid change in the mycobacterial lipid transporter MmpL3 from V643 to M. Amino acid changes in V643 or neighboring F644 have previously been reported in the context of spontaneous resistance mutants of other putative MmpL3 inhibitors with distinctly different chemical scaffolds (Ioerger et al., 2013; Remuinan et al., 2013; Zheng et al., 2018) (Supplementary Figure 3). As our mutant selection experiments were carried out using *M. bovis* BCG, E11 may have additional targets in Mtb that are absent in the non-pathogenic surrogate. MmpL3 orthologs of *M. bovis* BCG, *M. smegmatis* and *M. abscessus* share 99, 64, and 56% amino acid sequence identity with the Mtb ortholog, respectively. Although the tertiary structure of MmpL3 has not been reported, mycobacteria likely share functional motifs based on sequence identity, suggesting conserved modes of inhibition. Previous reports revealed an impact of amino acid changes in D640, Y641, and F644 on protein conformation and proton translocation (Belardinelli et al., 2016). A change in V643 located within this cluster and conferring E11 resistance may have a similar effect.

The *mmpL3* gene is essential for growth of Mtb *in vitro* and in mice (Li et al., 2016; Degiacomi et al., 2017) rendering it a target of interest for drug discovery. Recent studies demonstrated that MmpL3 is the flippase of mycobacteria translocating TMM from the inner leaflet to the outer leaflet of the IM (Xu et al., 2017). TMM is the precursor of TDM, one of the most abundant components of the mycobacterial cell envelope formed by enzymes of the Antigen 85 complex (Harth et al., 1996). We showed that E11 reduces TMM translocation across the IM in mycobacteria and consequently abolishes synthesis of new TDM (Figure 3). The use of spheroplasts of fast-growing *M. smegmatis* due to the unavailability of spheroplasts of slow-growing *M. bovis* BCG is a limitation of our study. E11 appears to have general non-specific effects on lipid biosynthesis that were more pronounced in *M. smegmatis* as compared to *M. bovis* BCG (Figures 3C,D) and require further studies to elucidate the underlying mechanism. Our genetic experiments provided evidence that MmpL3 is the cellular target of E11 and subsequent biochemical studies revealed that inhibition likely occurs by an indirect mechanism involving disruption of the proton motive force utilized by MmpL3 for TMM translocation. A surprisingly large number of putative MmpL3 inhibitors with diverse chemistry has been discovered in recent years: SQ109 and derivatives (Tahlan et al., 2012), BM212 (La Rosa et al., 2012), AU1235 (Grzegorzewicz et al., 2012), Indolecarboxamides (Rao et al., 2013), Indoleamides (Lun et al., 2013), Tetrahydropyrazole pyrimidine, and Spiro analogs (Remuinan et al., 2013), Compound 2 (Ioerger et al., 2013), C215 (Stanley et al., 2012), and HC2091 (Zheng et al., 2018). It has been suggested that several MmpL3 inhibitors may act indirectly by targeting the proton motive force, which drives MmpL3 lipid translocation (Li et al., 2014). The reason why MmpL3 appears so susceptible to small molecule inhibitors discovered in whole cell screens remains largely unclear. One may speculate that spontaneous resistance mutants identified in MmpL3 may be compensatory, masking inhibition of other cellular targets. For instance, spontaneous resistance mutant analysis initially suggested that Tetrahydropyrazole pyrimidine hits MmpL3 but more detailed analysis of the mechanism of action revealed that it targets the hydrolase, EchA6 (Cox et al., 2016). Similarly, it has been shown that SQ109 does not actually inhibit TMM flipping across the IM (Xu et al., 2017). More research is required to verify whether putative MmpL3 inhibitors indeed directly interact and inhibit MmpL3 function as demonstrated for BM212 (Xu et al., 2017).

The mycobacterial cell envelope proved to be a rich target space for drug discovery and it has been clinically validated. Our cell envelope stress reporter assay utilizing the *iniBAC* promoter is a versatile screening tool to tap on this target space. The combined screening approach introduced here successfully identified the novel acetamide E11, which inhibits mycolic acid transport. A series of E11 derivatives showing a dynamic structure-activity relationship may guide further optimization and subsequent testing in animal models to strengthen the development pipeline of urgently needed antimycobacterials.

## Data Availability Statement

All relevant data sets analyzed for this study are included in the manuscript and the supplementary files.

## Authors Contributions

AS, AP, YY, TD, and MG conceived the project and designed the strategy. AS, ZX, UL, JH, MC, and YY performed experiments. AS, ZX, S-SC, AP, TD, and MG analyzed the data and wrote the manuscript.

### Conflict of Interest Statement

The authors declare that the research was conducted in the absence of any commercial or financial relationships that could be construed as a potential conflict of interest.
