# Supplementary material for: Novel Acetamide Indirectly Targets Mycobacterial Transporter MmpL3 by Proton Motive Force Disruption
Source: Front Microbiol. 2018 Dec 4;9:2960. doi: 10.3389/fmicb.2018.02960 (PMC6289072; doi:10.3389/fmicb.2018.02960)
Supplement: Supplementary file 1 [file Table_1.pdf]

Supplementary Material

Novel acetamide targeting mycobacterial transporter MmpL3

Annanya Shetty, Zhujun Xu, Umayal Lakshmanan, Jeffrey Hill, Meng Ling Choong, Shu-Sin Chng, Yoshiyuki Yamada, Anders Poulsen, Thomas Dick, Martin Gengenbacher*

*** Correspondence:** Martin Gengenbacher, mg1435@njms.rutgers.edu

I. Supplementary Materials and Methods

TMM accessibility to LysB degradation with DDM solubilization

To examine if E11 affected the activity of LysB, samples were treated with DDM (final concentration 0.02% (w/v)) to lyse spheroplasts after radiolabeling and prior to LysB treatment. Following LysB treatment, lipids were extracted from the whole solution with a slightly modified procedure. Appropriate volumes of chloroform and methanol were added to the ~1-ml solution to give a single-phase chloroform-methanol-water (1:2:0.8) solution. The sample was then subjected to five repeats of alternating bath sonication (2 min duration) and brief vortexing (10 s). The final solution was converted to a two-phase chloroform-methanol-water (1:1:0.8) mixture by adding appropriate volumes of chloroform and water, vortexed and finally centrifuged at 4,000 x *g* for 30 min to achieve phase separation. The organic phases (bottom phase) containing lipids were collected and air-dried overnight in a chemical fume hood. Lipids were analyzed by TLC and visualized by phosphor imaging.

M. smegmatis spheroplast lysis assay

To test if treatment with E11 and LysB resulted in lysis of spheroplasts, mock reactions not containing radioactive sodium acetate were set up so as to examine cytoplasmic protein localization after cell fractionation. *M. smegmatis* mc^2155 spheroplasts (OD_{600} = 1) were pre-treated with DMSO or E11 for 15 min and incubated for 2 h at 37˚C in 1xSMM buffer (pH 6.8) containing sodium acetate (final concentration 18 µg/ml). 1-ml spheroplast suspensions were then aliquoted into separate microcentrifuge tubes and treated with purified LysB (50 µg/ml final concentration) or no LysB for 30 min at 37˚C. Treated spheroplasts were centrifuged at 5,000 x *g* for 10 min to separate cell pellets and supernatants. Pellets were washed twice and resuspended in 1-ml 1xSMM buffer (+ 150 mM NaCl). Supernatants were further purified by two sequential centrifugation steps (10,000 x *g*, 10 min) to remove residual spheroplasts. 100 µl from both pellet and supernatant samples were mixed separately with equal volumes of 2x Laemmli SDS-PAGE reducing sample buffer, and boiled at 100°C for 10 min. Equal amounts (normalized by ODs) of all samples were then subjected to SDS-PAGE, followed by immunoblotting using antibodies against GroEL2 (cytoplasmic protein) and the penta-histidine tag (to detect LysB-His).

Membrane potential measurements in *M. smegmatis* spheroplasts.

The effects of E11 on $\Delta\psi$ were determined using the membrane potential-sensitive 3,3'-dipropylthiodicarbocyanine ($DiSC_3(5)$) dye. $DiSC_3(5)$ binds to energized membranes and becomes quenched. When $\Delta\psi$ is disrupted, the dye leaves the membrane, resulting in an increase in fluorescence.

1.5-ml *M. smegmatis* spheroplasts (OD =0.8) were used in 1xSMM buffer containing 10 mM glucose and 1 µM nigericin (added to remove the effects of ΔpH). $DiSC_3(5)$ was then added to samples to get a final concentration of 5 µM and equilibrated for 10 min at room temperature. From this point, fluorescence was continuously monitored with a SPECTRAmax 250 microplate spectrophotometer equipped with SOFTmax PRO software (Molecular Devices), employing an excitation wavelength of 643 nm and an emission wavelength of 666 nm. The effects of E11 on Δψ were measured by monitoring increase in fluorescence when the compound was added at specific time points at indicated concentrations.

II. Supplementary Figures

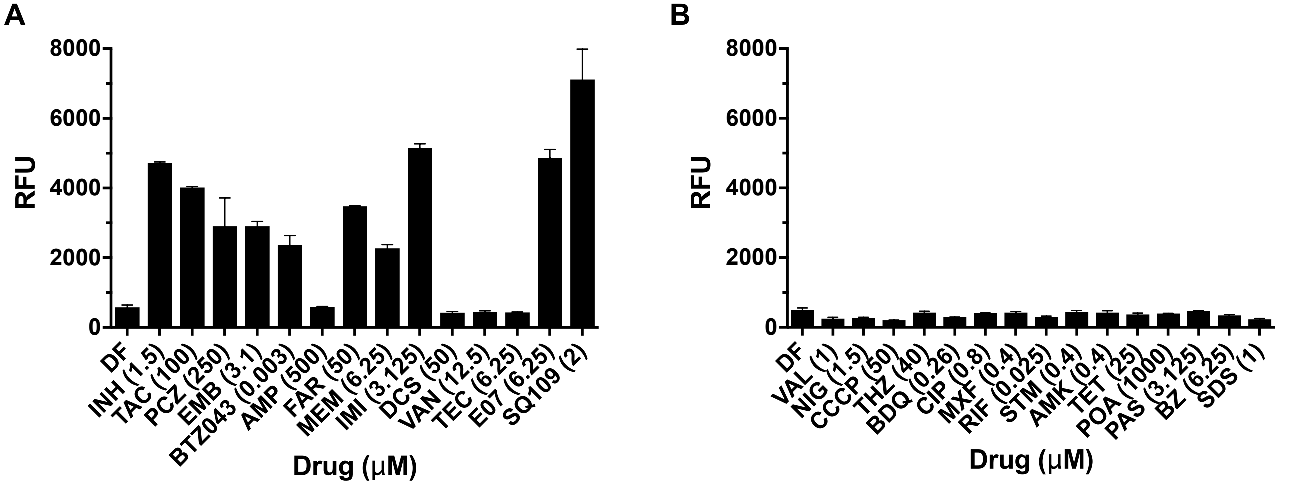

Supplementary Figure 1. Cell wall targeting antibiotics induce *M. bovis* BCG p*iniBAC*-RFP reporter strain. A panel of antibacterials covering most classes was used to test for their ability to induce cell wall stress measured by induction of the *M. bovis* BCG p*iniBAC*-RFP reporter strain at $1\times MIC_{90}$. After 24 h of incubation in the presence of each drug, red fluorescence emitted by the reporter was determined and shown as Relative Fluorescence Units (RFU). **(A)** INH, isoniazid; TAC, thiacetazone; PCZ, perchlozone; EMB, ethambutol; BTZ043, benzothiazinone; AMP, ampicillin; FAR, faropenem; MEM, meropenem; IMI, imipenem; DCS, D-cycloserine; VAN, vancomycin; TEC, teicoplanin, E07 (Table 1) , indolecarboxamide derivative and ethylenediamine SQ109. **(B)** VAL, valinomycin; NIG, nigericin; CCCP, carbonyl cyanide m-chlorophenyl hydrazine; THZ, thioridazine; BDQ, bedaquiline; CIP, ciprofloxacin; MXF, moxifloxacin; RIF, rifampicin; STM, streptomycin; AMK, amikacin; TET, tetracycline; POA, pyrazinoic acid; PAS para-amino salicylic acid; BZ, bortezomib and SDS, sodium dodecyl sulfate. Experiments were performed twice with duplicate samples. Data is represented as means and standard deviations. DF, drug free.

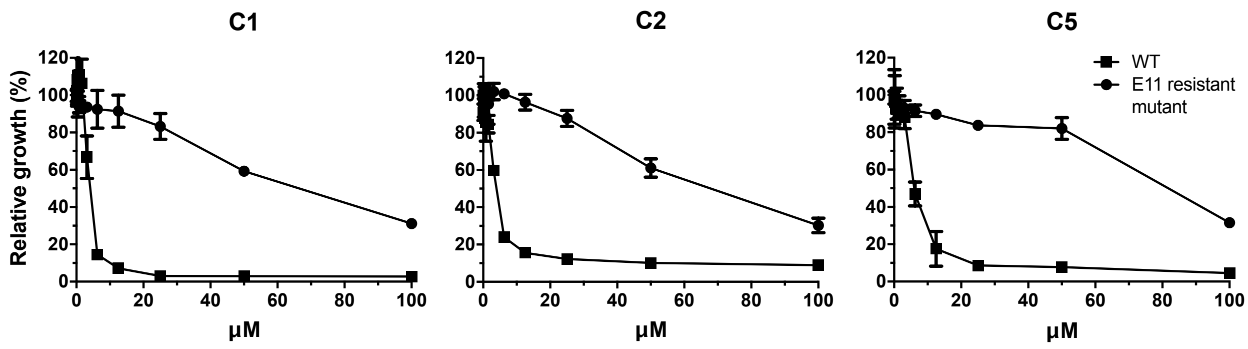

Supplementary Figure 2. Dose response profile of spontaneous E11 resistance mutants. In three different experiments, three spontaneous *M. bovis* BCG mutants (C1, C2 and C5) were selected on agar supplemented with E11. Growth inhibition of the three mutants at a range of E11 concentrations relative to *M. bovis* BCG wildtype is shown. The MIC_{90}s of C1, C2 and C5 were > 100 µM, indicating a more than 8-fold increase as compared to the wildtype control. Each mutant was independently profiled three times. Mean values and standard deviations are shown.

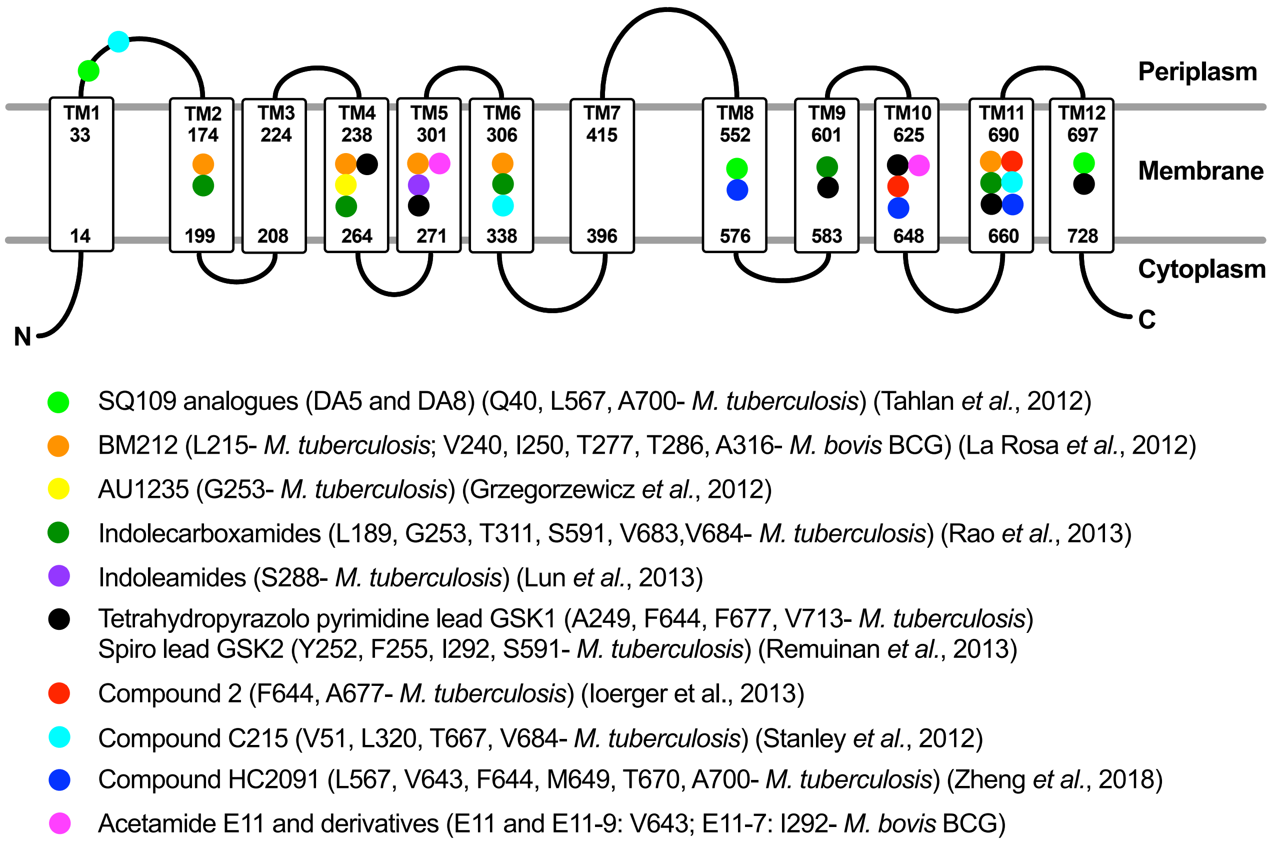

Supplementary Figure 3. Predicted topology of MmpL3 and drug-resistance conferring amino acid residues. Depicted are the mutated amino acid residues in MmpL3 of *M. bovis* BCG and Mtb that confer resistance to known MmpL3 inhibitors including acetamide E11 and its derivatives, E11-7 and E11-9 of this study. Topology adopted from Belardinelli *et al.*, 2016.

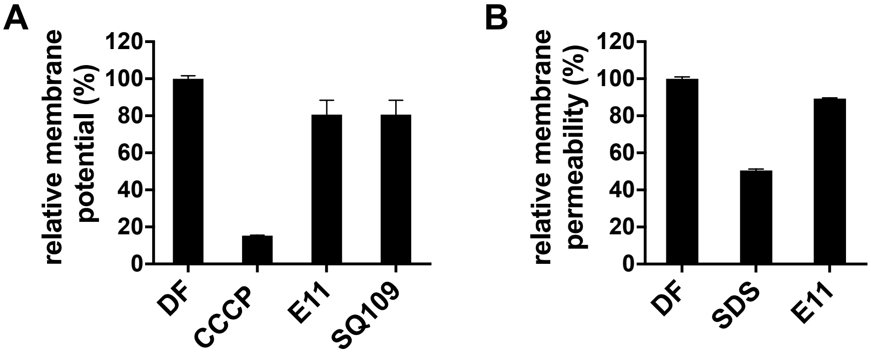

Supplementary Figure 4. E11 does not affect membrane potential and permeability of mycobacteria. (A) *M. bovis* BCG cultures were treated with 50 µM (1×MIC_{90}) of membrane potential disrupting drug carbonyl cyanide m-chlorophenyl hydrazone (CCCP), 50 µM (4×MIC_{90}) E11 and 6 µM (4×MIC_{90}) SQ109 for 24 h. The membrane potential relative to the drug-free control was plotted. **(B)** *M. bovis* BCG cultures were treated with 5% sodium dodecyl sulfate (SDS) and 50 µM (4×MIC_{90}) E11 for 24 h and permeabilized bacilli relative to the drug-free control are shown. The data is representative of three independent experiments.

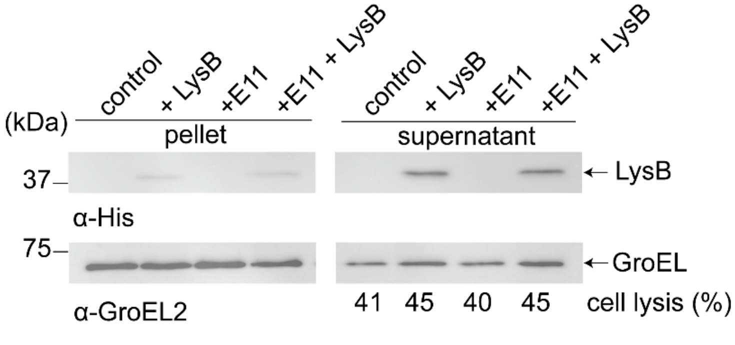

Supplementary Figure 5. E11 did not result in the cell lysis of *M. smegmatis* spheroplasts. α-GroEL2 and α-His immunoblot analyses of pellet and supernatant fractions obtained from sedimentation of *M. smegmatis* spheroplasts in the presence of DMSO and E11 (4×MIC), following treatment with purified LysB. Quantification of bands in the supernatants, compared to the corresponding pellets averaged around three experiments, indicate ~38-43% cell lysis across the experiment.

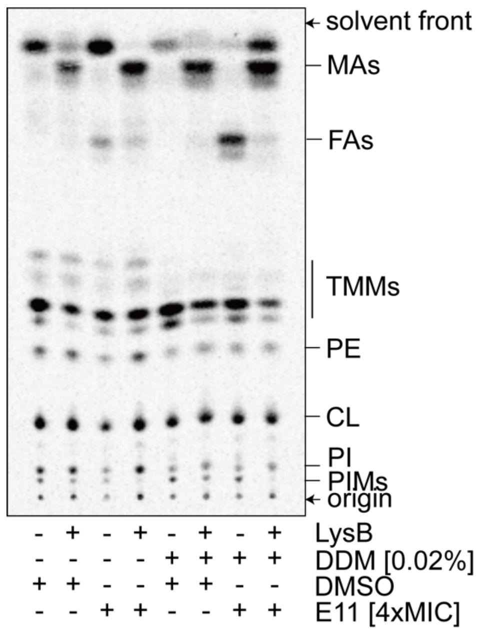

Supplementary Figure 6. E11 does not affect the activity of LysB. Representative TLC analysis of [^{14}C]-labelled lipids newly-synthesized in the presence of indicated concentrations of E11, and extracted from *M. smegmatis* spheroplasts following treatment with or without purified LysB. Where indicated, n-dodecyl-β-maltoside (DDM) was added to solubilize spheroplasts immediately prior to LysB addition. DMSO was used to dissolve the compounds and thus serve as negative controls. Equal amounts of radioactivity were spotted for each sample. The developing solvent system comprised of chloroform-methanol-water (30:8:1). The percentage of TMMs accessible to LysB is given by the difference in TMM levels between samples with or without LysB treatment, normalized against control samples without LysB treatment. TMM levels in each sample were quantified as a fraction of total mycolates (TMM+MA). FA, fatty acid.

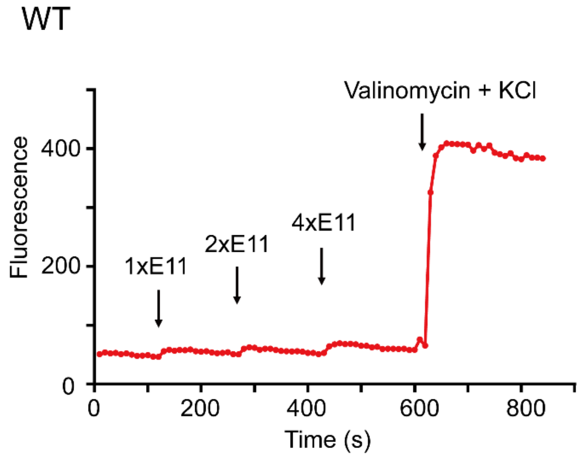

Supplementary Figure 7. E11 has minimal effect on the membrane potential (Δψ) in *M. smegmatis* spheroplasts. Fluorescence intensity changes of 3,3'-dipropylthiadicarbocyanine iodide ($DiSC_3(5)$) dye in spheroplasts upon the addition of specified concentrations of E11 at the indicated time points. $DiSC_3(5)$ binds to energized membranes and becomes quenched. When Δψ is disrupted, the dye leaves the membrane, resulting in an increase in fluorescence. Valinomycin-K^+ serves as positive control.

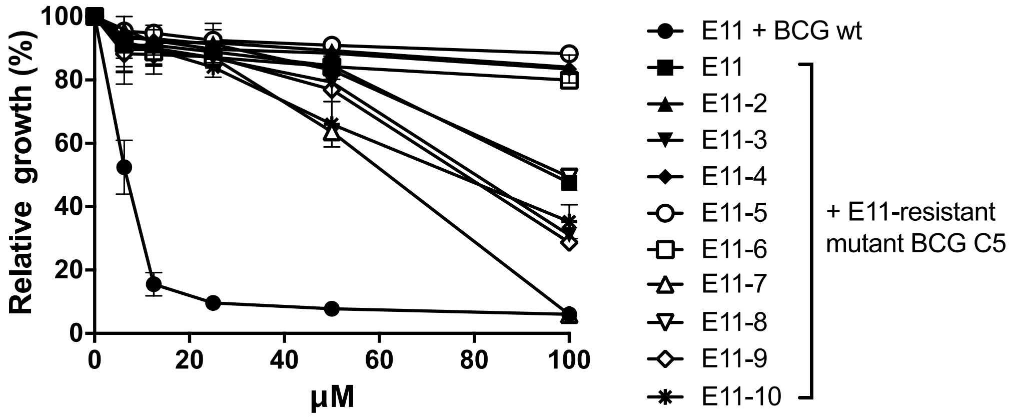

Supplementary Figure 8. E11 analogs are cross resistant to spontaneous E11 *M. bovis* BCG mutant C5. Growth inhibition of mutant C5 (**Supplementary Figure 2**) was determined relative to *M. bovis* BCG wildtype using 2-fold dilutions of E11 and derivates. MIC_{50} values of all test compounds were > 50 µM suggesting cross resistance and on-target activity. Means and standard deviations shown are representative of three independent experiments performed in duplicate.

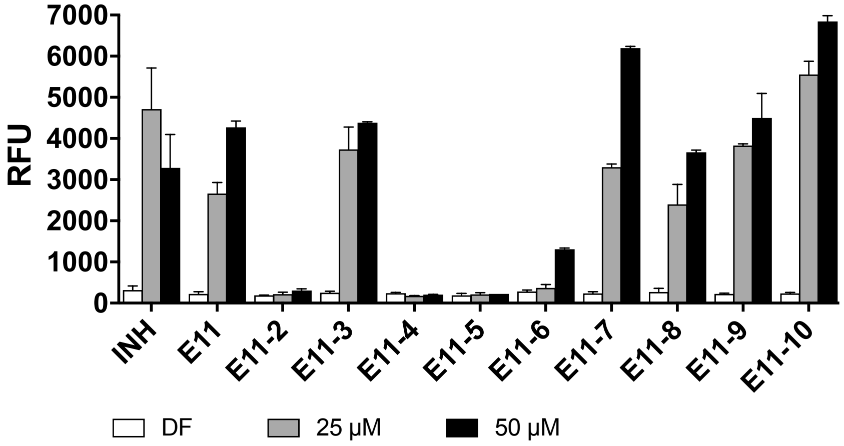

Supplementary Figure 9. Capacity of E11 analogs to induce cell wall stress. The acetamide compound series E11-2 to E11-10 was tested at 25 µM and 50 µM concentration using the *M. bovis* BCG p*iniBAC*-RFP reporter strain. Fluorescence signals measured after 24 h of incubation demonstrate a correlation between p*iniBAC*-induction and *in vitro* potency (**Table 2**). Data is represented as mean and standard deviation representative of three independent experiments.
